# The plasma membrane H^+^‐ATPase, a simple polypeptide with a long history

**DOI:** 10.1002/yea.3365

**Published:** 2018-12-10

**Authors:** Michael Palmgren, Pierre Morsomme

**Affiliations:** ^1^ Department of Plant and Environmental Sciences University of Copenhagen Frederiksberg C Denmark; ^2^ Louvain Institute of Biomolecular Science and Technology (LIBST) UCLouvain Louvain‐la‐Neuve Belgium

**Keywords:** Arabidopsis thaliana, F‐type ATPase, *Neurospora crassa*, Nicotiana tabacum, proton pump, P‐type ATPase, Saccharomyces cerevisiae, *Schizosaccharomyces pombe*

## Abstract

The plasma membrane H^+^‐ATPase of fungi and plants is a single polypeptide of fewer than 1,000 residues that extrudes protons from the cell against a large electric and concentration gradient. The minimalist structure of this nanomachine is in stark contrast to that of the large multi‐subunit F_O_F_1_ ATPase of mitochondria, which is also a proton pump, but under physiological conditions runs in the reverse direction to act as an ATP synthase. The plasma membrane H^+^‐ATPase is a P‐type ATPase, defined by having an obligatory phosphorylated reaction cycle intermediate, like cation pumps of animal membranes, and thus, this pump has a completely different mechanism to that of F_O_F_1_ ATPases, which operates by rotary catalysis. The work that led to these insights in plasma membrane H^+^‐ATPases of fungi and plants has a long history, which is briefly summarized in this review.

… This raises a fundamental question. Why are the two presumed proton pumps of the yeast cell, the mitochondrial and the plasma membrane ATPases, so different in their structure and mechanism? … A valid answer to the apparent paradox raised by the existence of two distinct proton pumps in yeast requires definite elucidation of another question. Does the plasma membrane ATPase from yeast really pump protons and only protons? Amory, Foury, and Goffeau (1980)

From time to time, surprise has been expressed that the proton‐motive F_O_F_1_ ATPases should be so different structurally from the cation‐motive E_1_E_2_ ATPases, and that their (chemiosmotic) mechanisms should presumably be so different inasmuch as the E_1_E_2_ ATPases involve a phosphorylated intermediate while the F_O_F_1_ ATPases do not. Mitchell and Koppenol (1982)

## 1 INTRODUCTION

It has been known since ancient times that yeast secretes acid during glucose fermentation without this phenomenon being connected with any essential process in yeast. This review recapitulates the discovery of the plasma membrane proton pump (H^+^‐ATPase) and will highlight recent advances in our understanding of the function, regulation, and structure of this pump, so surprisingly different from other proton pumps that it caused eyebrows to rise (as the introductory quotes indicate). The history to be described is divided into several parts, the biophysical, the biochemical, and the molecular biology approaches. Here, only a brief account is given, which is focused and dedicated to the work and memory of Professor André Goffeau. For a more detailed account of the early history, the reader is referred to Goffeau and Slayman (1981). Subsequently, some mention will be made to the history of the corresponding enzyme in plants.

In the late 1960s, the chemiosmotic hypothesis of Peter Mitchell won acceptance and, with the words of Efraim Racker (1975), “became a favorite tool in the design of experiments.” Oxidative phosphorylation of ADP to generate ATP had been shown to be carried out by the F_O_F_1_ ATPase, a large mitochondrial complex, which as an energy source utilizes a proton motive force that has two components: a proton gradient and a membrane potential. In the 1970s, it became increasingly clear that the plasma membrane of fungi and plants is equipped with a system running in the reverse direction, thus operating as an ATP‐driven proton pump that generates a proton motive force.

## 2 HISTORY OF THE PHYSIOLOGY OF FUNGAL PLASMA MEMBRANE H^+^‐ATPases

The father of the plasma membrane H^+^‐ATPase is Clifford L. Slayman who, using inserted microelectrodes, recognized that cells of the fungus *Neurospora crassa* have a substantial plasma membrane potential approximating a quarter of a volt. Inhibitors of ATP synthesis caused depolarization of the membrane, suggesting that formation of the membrane potential depended on ATP consumption (Slayman, 1965a, 1965b; Slayman, Long, & Lu, 1973; Slayman, Lu, & Shane, 1970). It was suggested that the membrane potential is sustained by ejection of protons coupled to the splitting of ATP. This membrane potential then serves as the main energy distributor for transport and—together with an inward chemical gradient for protons—drives the uptake of a variety of different substances (Slayman & Gradmann, 1975). Protons were hypothesized to be the transported ion because hydrogen ions, almost alone among the common inorganic cations and anions, had a steep depolarizing effect: 30–40 mV for each unit decrease of pH (Slayman & Gradmann, 1975). This hypothesis has now proven to be correct; however, at the time, it was not supported by any biochemical or molecular evidence. Prokaryotes have plasma membrane‐localized F_O_F_1_ ATPases. Therefore, even if the presence of a proton pump in the plasma membrane could be demonstrated, it needed not to be unrelated to the F_O_F_1_ ATPase of mitochondria.

An important characteristic of the F_O_F_1_ ATPase is that it is sensitive to the antibiotic oligomycin (Lardy, Johnson, & McMurray, 1958); in fact, the F_O_ in F_O_F_1_ received its name for being the oligomycin‐sensitive factor (Racker, 1963). Already in 1967, it was reported that the plasma membrane of the yeast Saccharomyces cerevisiae contained an ATPase activity that was insensitive to oligomycin (Matile, Moor, & Mühlethaler, 1967). In situ freeze‐etching and subsequent negative staining by phosphotungstate of yeast cells revealed that the outer surface of the plasma membrane was covered with globular particles of about 150 Å in diameter. To purify plasma membranes, a microsomal membrane preparation was centrifuged through a linear density gradient of urografin, and a distinctive white band was isolated in which the membranes were sculptures with particles of the same size. It was thus concluded that this membrane fraction represented the plasma membrane. Matile et al. (1967) found that the most remarkable constituent of the plasma membrane was a polysaccharide identified as mannan, and the activity of the single enzyme they could detect, an ATPase. The ATPase was dependent on Mg^2+^ for activity but was completely insensitive to the mitochondrial ATPase inhibitor oligomycin. It was stated:

No direct evidence with regard to the functional meaning of the ATPase can be presented yet. It is likely that this enzyme is involved in the energy‐dependent uptake of nutrients from the culture medium; that is, permeases such as amino acid absorbing enzymes which are present in yeast cells may be identical with the ATPase bound to the plasma membrane.

The hypothesis that nutrient uptake systems were directly energized by ATP was prevalent at that time.

It had been suggested before that H^+^ secretion and/or ATP was required for uptake of nutrients into fungal cells. Conway and O'Malley (1946) observed that addition of K^+^ to the medium of fermenting yeast cells increased secretion of H^+^ and noted:

The rapid absorption of K is essentially an interchange with H ions, as shown by the practically quantitative relations of K absorbed and H extruded … It would appear that the nature of the potassium absorption consists very largely in a direct exchange of K and H ions.

Peña, Cinco, Puyou, and Tuena (1969) studied the effect of K^+^ on the respiration and glycolysis of yeast cells and noted:

Measurement of the levels of ADP, ATP and Pi revealed that K^+^ stimulates an enzymatic activity that diminishes ATP and increases ADP... This might mean that the ion induces the breakdown of ATP by an indirect mechanism, with another phosphorylated compound being hydrolyzed directly … The most obvious condition of this sort would be the pH increase created inside the cell as H^+^ is exchanged for K^+^.”

Similarly*,* Eddy, Indge, Backen, and Nowacki (1970) noted that glycine uptake into yeast cells was markedly increased when pH of the medium was lowered from pH 7 to pH 4.5. This pH dependence was more marked when glucose was absent than when it was present. In contrast, high external K^+^ inhibited glycine uptake. This led them to suggest:

The observations lead to the idea that, in certain circumstances, H^+^ and K^+^ may play part in the transport of amino acids by yeast that is analogous to the roles of Na^+^ and K^+^ in mammalian systems.

In these early studies, there was no suggestion that nutrient uptake could be a process indirectly fueled by metabolic energy, and there was no mentioning of an ATPase or a H^+^ pump.

Foury and Goffeau (1975) observed a correlation between all three parameters: ATPase activity, extrusion of protons, and uptake of nutrients. They found that Dio‐9, an F_O_F_1_ ATPase inhibitor (Schatz, Penefsky, & Racker, 1967), reduced intracellular ATP levels and instantaneously suppressed the cellular ejection of protons as well as the uptake of uridine and amino acids. However, Dio‐9 also inhibited proton extrusion in the presence of antimycin A, an inhibitor of respiration. Furthermore, in the presence of antimycin A, addition of glucose to glucose‐starved cells increased ATP levels and proton extrusion. This led Foury and Goffeau (1975) to conclude:

These results suggest that in these conditions, the target of Dio‐9 is not the mitochondrial ATPase but a plasma membrane proton‐translocating function generating an electrochemical gradient required for active transport.

## 3 HISTORY OF THE BIOCHEMISTRY OF FUNGAL PLASMA MEMBRANE H^+^‐ATPases

The heroes who biochemically characterized the plasma membrane ATPase, demonstrated that it was a P‐type ATPase and not an F_O_F_1_ ATPase, and finally proved it to be a proton pump were many, but prominent names were André Goffeau (working on *Schizosaccharomyces pombe*), Ramon Serrano (working on Saccharomyces cerevisiae), and Gene Scarborough (working on *Neurospora crassa*).

To characterize the elusive enzyme, plasma membranes first had to be purified with minimal contamination of mitochondrial membranes having F_O_F_1_ ATPase activity. This was not a trivial task as no marker for the plasma membrane was available at the time. Gene Scarborough solved this problem by employing a wall‐less mutant of N. crassa that was labeled on the outside with concanavalin A, which binds to carbohydrates on the outer membrane surface. Cells were then disrupted, and a concanavalin A‐labeled membrane fraction was obtained. The plasma membrane‐enriched fraction contained a major membrane‐embedded enzyme that hydrolyzed ATP and was stimulated by Na^+^ and K^+^ (Scarborough, 1975). Stimulation by these cations is indicative of Na^+^/K^+^‐ATPase, but Scarborough was careful not to suggest any ligand specificity of the ATPase. Scarborough subsequently demonstrated that the isolated plasma membrane vesicles accumulated thiocyanate ion (SCN^−^) when ATP was added. As SCN^−^ is a membrane permeable anion, the result of the experiment suggested that, when ATP was hydrolyzed, a membrane potential, positive on the inside, was generated across the vesicle membrane (Scarborough, 1976). It was concluded that the plasma membrane ATPase of N. crassa is electrogenic, albeit the transported ligand was still unidentified. The N. crassa enzyme was subsequently characterized in more detail by Bowman and Slayman (1977; the last author was Carolyn W. Slayman). Following purification of plasma membranes by the same procedure as that of Scarborough, they demonstrated that the N. crassa plasma membrane ATPase activity is insensitive to the F_O_F_1_ ATPase inhibitor oligomycin and has a pH optimum of 6, in contrast to the pH optimum of the F_O_F_1_ ATPase of N. crassa, found to be pH 8.25. This work demonstrated that N. crassa has an ATPase in its plasma membrane that is different in properties from the F_O_F_1_ ATPase of mitochondria.

The same characteristics were soon thereafter reported for the plasma membrane ATPase of the yeasts *S. pombe* (Delhez, Dufour, Thines, & Goffeau, 1977) and S. cerevisiae (Serrano, 1978). Delhez et al. (1977) separated microsomal membrane vesicles by centrifugation on a sucrose density gradient and obtained two peaks with ATP hydrolytic activity. Membrane vesicles in the fraction with the highest density were stained with osmium tetraoxide, which is indicative of mannose and thus polysaccharides, a characteristic of the outer surface of the plasma membrane. The ATPase in the plasma membrane‐enriched fraction had a pH optimum around pH 5.3, far more acidic than the pH optimum of 9 in the lighter fraction, which is characteristic of the F_O_F_1_ ATPase (Delhez et al., 1977). Serrano (1978) used a very similar purification strategy for isolating S. cerevisiae plasma membranes and tested their plasma membrane origin by postfixation in osmium tetroxide and staining with uranyl acetate and lead citrate. The plasma membrane fraction contained an ATPase activity with a pH optimum of 5.4 that was insensitive to oligomycin.

The work of Scarborough had established that there was an electrogenic ATPase at the plasma membrane of N. crassa, but the polypeptide(s) responsible for the process had not been identified. Characterization of the plasma membrane ATPase first required its purification in a catalytically active form. Using the mild detergent lysophosphatidylcholine, Dufour and Goffeau (1978) were able to solubilize the *S. pombe* plasma membrane fraction, and after subsequent centrifugation of the solubilized membrane proteins through a linear 10% to 30% (*w*/w) sucrose gradient, the ATPase activity became separated from the bulk of contaminating proteins, which were of lower sedimentation rate. The plasma membrane ATPase peak fraction of the sucrose gradient was analyzed by SDS‐polyacrylamide gel electrophoresis followed by Coomassie blue staining. Only a single polypeptide with an apparent molecular weight of 105,000 could be observed. Thus, the purified plasma membrane ATPase of *S. pombe* exhibited a much simpler subunit composition than the mitochondrial, chloroplast, or bacterial ATPases. In contrast, it appeared very similar in size to the catalytic subunit of mammalian Na^+^/K^+^‐stimulated plasma membrane and Ca^2+^‐stimulated sarcoplasmic reticulum ATPases. These enzymes were at that time called E_1_E_2_ ATPases because during catalysis, they appeared to alternate between two major conformations, Enzyme 1 (E_1_) and Enzyme 2 (E_2_; Siegel & Albers, 1967). The plasma membrane ATPase of S. cerevisiae could not be solubilized in an active form with lysophosphatidylcholine, but could be solubilized with a synthetic zwitterionic detergent and subsequently purified by centrifugation through a linear glycerol gradient (Malpartida & Serrano, 1980). In the fraction with highest ATP hydrolytic activity, 85% of the protein corresponded to a 105,000‐Mw polypeptide, thus confirming the single subunit composition of the plasma membrane ATPase. Subsequently, the N. crassa ATPase was purified and shown to have similar characteristics as the *S. pombe* and S. cerevisiae enzymes (Bowman, Blasco, & Slayman, 1981).

E_1_E_2_ ATPases are now commonly called P‐type ATPases because phosphorylation of a conserved aspartate residue initiates pumping during catalysis. Before classification of the plasma membrane ATPase as a P‐type ATPase, it was essential to demonstrate that a phosphorylated intermediate was formed. Willsky (1979) analyzed a crude microsomal fraction from S. cerevisiae and found that at pH 5.5 (optimum pH of the plasma membrane H^+^‐ATPase), three polypeptides at 210,000, 160,000, and 115,000 molecular weight, respectively, were labeled with [γ‐^32^P]ATP. All phosphoproteins behaved as phosphorylated intermediates of E_1_E_2_ ATPases. Thus, they formed rapidly in the presence of Mg^2+^, there was a rapid turnover of the bound phosphate when unlabeled ATP was added, and dephosphorylation occurred after incubation with hydroxylamine, which is indicative that the phosphate linkage is a carboxylic acid anhydride linkage (Lipmann & Tuttle, 1945). In addition, vanadate, an inhibitor of E_1_E_2_ ATPase activity, blocked the phosphorylation of the 210,000‐ and 115,000‐Da proteins. Serrano and Malpartida (1979) carried out similar experiments with a plasma membrane fraction from S. cerevisiae and found a 100,000‐Mw band to be radiolabeled by ^32^P‐ATP. Using N. crassa plasma membranes, Dame and Scarborough (1980) similarly found that, following phosphorylation by ^32^P‐ATP, a radiolabeled protein migrated in gels with an apparent molecular weight of 100,000. The radiolabel disappeared if the membranes had been pretreated with trypsin but was protected if incubation was in the presence of MgATP. SDS polyacrylamide gel electrophoresis followed by Coomassie Blue staining showed that a polypeptide with an apparent molecular weight of 105,000 was cleaved by trypsin but was protected if MgATP was present during proteolysis. Strikingly, the ATP hydrolytic activity of the plasma membranes was sensitive to trypsin treatment, but not in the presence of MgATP. This served as a strong indication that the radiolabeled polypeptide indeed corresponded to a 100,000‐Mw ATPase. Dame and Scarborough (1981) further characterized the chemical nature of the phosphoryl enzyme intermediate of the phosphorylated N. crassa plasma membrane enzyme and demonstrated that it was an aspartylphosphate, identical to that of the Na^+^/K^+^‐ATPase and Ca^2+^‐ATPase of animal cells.

Still, the identity between the radiolabeled protein and the plasma membrane ATPase remained uncertain. Final proof for a phosphorylated reaction cycle intermediate of the plasma membrane ATPase came when the purified 100,000‐Mw plasma membrane ATPase from *S. pombe* was shown to form a phosphorylated reaction cycle intermediate (Amory et al., 1980). The phosphorylated intermediate reached the steady‐state level in less than 2 s and rapidly turned over. The phosphobond was cleaved by hydroxylamine and was relatively stable in acids but readily hydrolyzed in alkaline or in acid alcoholic media. These results demonstrated that the intermediate was an acylphosphate. Similar results were subsequently obtained with the purified S. cerevisiae enzyme (Malpartida & Serrano, 1981a).

Dame and Scarborough (1980), still using a plasma membrane preparation, used two independent methods to demonstrate that the ATPase of N. crassa plasma membranes is a proton pump. This was not a straightforward task as protons cannot be labeled and a demonstration of their transport across a membrane required that they could be measured on one side of a membrane or the other. First, isolated N. crassa plasma membrane vesicles were shown to catalyze concentrative uptake of the probe [^14^C] imidazole, which is membrane permeant in its unprotonated form but remains trapped into vesicles when protonated. MgATP‐dependent imidazole uptake and MgATP‐dependent membrane potential generation (measured as SCN^−^ uptake) displayed identical saturation kinetics with respect to the concentration of MgATP and were inhibited in parallel by the P‐type ATPase inhibitor orthovanadate. Furthermore, the addition of ATP (in the presence of Mg^2+^) to vesicles containing fluorescein‐labeled dextran, which is a pH indicator in the pH range between 5 and 8, gave rise to time‐dependent fluorescence quenching that was markedly stimulated by the addition of SCN^−^ and was abolished by orthovanadate. Taken together, these experiments demonstrated that the N. crassa ATPase was an electrogenic enzyme catalyzing H^+^ transport.

What remained to be shown was whether the purified single polypeptide plasma membrane ATPase was sufficient for proton pumping. In principle, many more polypeptides (subunits) or accessory proteins present in the plasma membrane preparation could be required for the process. This could be addressed by reconstituting the purified protein into artificial phospholipid bilayer vesicles and measuring ATP‐dependent transport of protons into the vesicle lumen. Methods for reconstituting the F_O_F_1_ ATPase had been developed by Efraim Racker in the late 1970s (Racker, Violand, O'Neal, Alfonzo, & Telford, 1979). Ramon Serrano had learned the technique during a postdoctoral stay in Racker's laboratory (Serrano, Kanner, & Racker, 1976) and reconstituted the purified plasma membrane ATPase of S. cerevisiae by a freeze‐thaw sonication procedure (Malpartida & Serrano, 1981b). Proton pumping was subsequently measured by an indirect approach. The idea, which had been tested on the F_O_F_1_ ATPase (Serrano et al., 1976), was that, once a steep proton gradient had been established, the pump would operate in reverse and catalyze ATP synthesis. Conditions were established to measure ^32^Pi‐ATP exchange, during which ^32^Pi is incorporated into cold ATP, and testing whether the reaction was sensitive to protonophores and other ionophores. Surprisingly, the reaction was only partly sensitive to the addition of the proton ionophore (uncoupler) carbonyl cyanide *m*‐chlorophenyl hydrazine (CCCP), but was further inhibited by addition of the potassium ionophore valinomycin, which by itself was ineffective. This made it difficult to conclude whether the enzyme was an electrogenic H^+^ pump or an electroneutral H^+^/K^+^ exchanger (Malpartida & Serrano, 1981b). In a subsequent publication (Malpartida & Serrano, 1981c), this view was modified in favor of transport of H^+^ only, as another proton ionophore, 1799, was shown to completely inhibit the energy transfer reaction. Furthermore, ATP‐dependent acidification of the vesicle lumen was documented by quenching of fluorescence of the acrididine dye 9‐amino‐6‐chloro‐2‐methoxiacridine (ACMA), which had previously been employed to monitor pH gradients (acidic inside) in submitochondrial particles (Azzi, Fabbro, Santato, & Gherardini, 1971).

Conclusive proof that the purified 105,000‐Mw polypeptide operates on its own as an electrogenic H^+^‐ATPase came following reconstitution of the *S. pombe* enzyme into lipid vesicles (Villalobo, Boutry, & Goffeau, 1981). Proton movement in the external medium was directly monitored with a pH‐electrode. Upon addition of MgATP, a fast proton uptake took place when countertransport of K^+^ (for charge compensation) was facilitated by inclusion of the K^+^ ionophore valinomycin. That the observed H^+^ translocation only took place in the presence of valinomycin was evidence of the electrogenic nature of the H^+^ pumping by the ATPase. Moreover, during steady‐state ATP hydrolysis, a fast and transient proton uptake was also observed when the generated membrane potential was collapsed upon addition of valinomycin in the presence of K^+^. The ATPase activity of the reconstituted enzyme was strongly stimulated by the H^+^‐conducting agent CCCP or by an association of the K^+^/H^+^ carrier nigericin plus valinomycin in the presence of K^+^.

An important discovery that demonstrated that the plasma membrane H^+^‐ATPase is under tight metabolic control came when it was shown that the enzyme is posttranslationally activated by glucose metabolism in a way that increases proton pumping tenfold (Serrano, 1983).

## 4 HISTORY OF THE MOLECULAR BIOLOGY OF FUNGAL PLASMA MEMBRANE H^+^‐ATPases

The heroes entering the new era of molecular biology of plasma membrane H^+^‐ATPases were again André Goffeau (working on *S. pombe*), Ramon Serrano (working on S. cerevisiae), and Carolyn W. Slayman (working first on N. crassa and later on S. cerevisiae).

The plasma membrane H^+^‐ATPase gene *Plasma Membrane ATPase1* (*PMA1*) was first cloned from S. cerevisiae by Serrano, Kielland‐Brandt, and Fink (1986), who could also show that the gene was essential for growth of yeast, and subsequently the corresponding gene was cloned from N. crassa (Addison, 1986; Hager et al., 1986) and *S. pombe* (Ghislain, Schlesser, & Goffeau, 1987). A second gene, *PMA2*, was identified in S. cerevisiae (Schlesser, Ulaszewski, Ghislain, & Goffeau, 1988) and *S. pombe* (Ghislain & Goffeau, 1991). Analysis of the sequences proved that the plasma membrane H^+^‐ATPase is indeed related to mammalian P‐type ATPases such as the Na^+^/K^+^‐ and the muscle Ca^2+^‐ATPases. With the gene sequences in hand, the door was open for genetic work and for structure–function analysis by mutagenesis.

A significant finding was that the glucose activation of the S. cerevisiae enzyme involved a short stretch of 11 C‐terminal residues that appeared to function as an autoinhibitor, which regulates the pump activity (Portillo, de Larrinoa, & Serrano, 1989). Later, it was found that glucose activation involved phosphorylation of two residues in this regulatory C‐terminal stretch (Lecchi et al., 2007; Lecchi, Allen, Pardo, Mason, & Slayman, 2005).

Soon, the importance of other residues for functioning of the yeast pump was investigated by site‐directed mutagenesis (Serrano & Portillo, 1990). However, studying in detail loss‐of‐function mutants was a problem, as the yeast plasma membrane H^+^‐ATPase is an essential enzyme and the cells would stop growing if the pump was not active (Serrano et al., 1986). This problem was solved by designing expression systems in which the endogenous wild‐type plasma membrane H^+^‐ATPase was put under the control of an inducible promoter (Cid, Perona, & Serrano, 1987) or in which mutant H^+^‐ATPases accumulated in secretory vesicles (Nakamoto, Rao, & Slayman, 1991). However, a problem that still remained was that mutations of many conserved residues gave rise to misfolded or mistargeted mutant proteins (DeWitt, Santos, Allen, & Slayman, 1998; Nakamoto et al., 1998), which complicated their analysis. For an in‐depth review on the structure, function, and biogenesis of the yeast plasma membrane H^+^‐ATPase, the reader is referred to Morsomme, Slayman, and Goffeau (2000).

## 5 A FOLLOW UP ON THE PLANT PLASMA MEMBRANE H^+^‐ATPase

It was suggested already in the beginning of the 20th century that ion uptake in plants was an active process distinct from diffusion. For reviews on the early history of the study of ion transport in plants, the reader is referred to Higinbotham (1973a, 1973b), Poole (1978), and Palmgren (1998).

At about the same time as the chemiosmotic hypothesis for ATP synthesis in mitochondria was proposed by Mitchell (Mitchell, 1961), Jack Dainty (1962) in a review discussed the presence of a plasma membrane potential in plants and their importance for ion transport and discussed whether ion pumps could be responsible for the electric potential. It is evident from the text that even the notion of a plasma membrane in plants was controversial at the time:

We may fairly ask the question: do the ion pumps make any direct contribution to the membrane potential? In the jargon of the specialists in this field: are any of the ion pumps electrogenic? Or are they neutral?... I do not propose to enter here into the controversy about the existence of the external plasma membrane, which I shall call the plasmalemma despite objections to the universal application of this term to the, presumed, external permeability barrier. I personally consider the evidence in its favor … I think this article should have shown that the proper study of ion transport in plant cells is in its infancy. Any investigation which is concerned only with ion concentrations is practically certain to lead to incorrect deductions because electrical potential gradients are just as important as concentration gradients in determining passive ion movements...

A problem with plant material for electrophysiological studies was that the cells are small, the vacuole is big, the existence of a plasma membrane was not even established, so it was difficult to be certain what was actually measured and across which membranes following insertion of the microelectrodes. To solve this problem, Spanswick, Stolarek, and Williams (1967) measured membrane potentials in the giant internodal cells of the macroscopic green alga Nitella translucens where it could be excluded that potential differences between the vacuole and its surroundings contributed to the recordings. As is evident from the conclusion, no mention is made about the possibility of a H^+^ pump:

The possibility of a contribution to the plasmalemma potential from electrogenic pumps is briefly discussed … This analysis shows that Na is actively transported from the cytoplasm into the medium as well as into the vacuole; K is pumped into the cytoplasm from the medium but appears to be close to electrochemical equilibrium across the tonoplast.

Inspired by the work of Slayman (1965a, 1965b), where the effect of external ions and metabolic inhibitors on the N. crassa membrane potential were studied, Kitasato (1968) used the metabolic inhibitor dinitrophenol (DNP) to study the origin of the membrane potential in N. translucens and became the first to obtain electrophysiological evidence pointing to a H^+^ extruding pump in plants. He concluded:

In artificial pond water containing DNP, the resting membrane potential decreased; this suggested that some energy‐consuming mechanism maintains the membrane potential at the resting level. It is probable that there is a H^+^ extrusion mechanism in the Nitella cell, because the potential difference between the resting potential and the H^+^ equilibrium potential is always maintained notwithstanding a continuous H^+^ inward current which should result from the potential difference … Perhaps the effect of DNP is the most conclusive evidence for a metabolic component of the observed membrane potential of the Nitella cell. Such a substance could not be expected to alter a phase boundary potential, but it could clearly be expected to uncouple phosphorylation in the cell and so decrease the energy stores available to an ionic pump.

A plant plasma membrane ATPase was first characterized by Hodges, Leonard, Bracker, and Keenan (1972). As a marker for the plant plasma membrane, they used a stain consisting of periodic acid, chromic acid, and phosphotungstic acid and could separate them from other membranes by discontinuous sucrose‐gradient centrifugation of homogenates from oat roots. An ATPase activity was found to copurify with the plasma membranes and was characterized (Hodges et al., 1972; Leonard & Hodges, 1973). They found the ATPase to be stimulated by multiple cations and suggested (Leonard & Hodges, 1973) that this single ATPase could be involved in the direct uptake of several ions:

In conclusion, purified plasma membranes of oat roots contain an ion‐stimulated ATPase that has kinetic properties similar to the kinetics of monovalent cation transport. This, along with other results discussed, provides strong evidence for a role for this ATPase in ion absorption by plants. The kinetic results presented here are also consistent with the concept that only one transport system, consisting of binding sites possessing varying affinities for ions, is involved in the absorption of ions from very low (0.01 mM) to very high (100 mM) concentrations.

Some of the very same scientists that had characterized the fungal plasma membrane H^+^‐ATPase now turned to plants to investigate whether the plasma membrane ATPases of plants could possibly be P‐type H^+^‐ATPases.

Vara and Serrano (1982) purified plasma membranes from oat roots using the method of Leonard and Hodges (1973), solubilized the ATPase from the plasma membranes, purified the activity, and found a concomitant enrichment of a polypeptide with an apparent molecular weight of 93,000. They then reconstituted the preparation into phospholipid vesicles using the freeze‐thaw sonication procedure used for the yeast enzyme, and, using ACMA as a probe for proton accumulation into the vesicular lumen, characterized the ATPase as an electrogenic proton pump stimulated by monovalent cations.

In a parallel line of work, Briskin and Leonard (1982a, 1982b) purified corn root plasma membranes and extracted them with deoxycholate to remove loosely attached polypeptides. In this preparation, they identified a polypeptide with an apparent molecular weight of 100,000 that formed a phosphorylated intermediate and was inhibited by vanadate as expected for a P‐type ATPase. The group of Goffeau (Scalla, Amory, Rigaud, & Goffeau, 1983) confirmed the presence of a 110,000 Mr polypeptide in a microsomal membrane preparation from corn roots and found it to be stimulated by monovalent cations and inhibited by vanadate like the plasma membrane H^+^‐ATPase of yeast. In a follow‐up study, Vara and Serrano (1983) corrected the apparent molecular weight of their partially purified oat root ATPase to be 105,000 and could demonstrate that it also formed a phosphorylated intermediate.

A purer plasma membrane H^+^‐ATPase preparation was obtained when lysophosphatidylcholine was used as a detergent to solubilize the oat root plasma membranes and when the ATPase was separated from contaminating proteins by glycerol density gradient centrifugation (Serrano, 1984). More than 70% of the protein in this ATPase‐enriched preparation was a polypeptide of about 100 kDa that formed a phosphorylated intermediate, was inhibited by vanadate, and could be reconstituted into phospholipid vesicles where it catalyzed electrogenic H^+^ transport.

Proteolytic digestion of the oat root plasma membrane H^+^‐ATPase situated in plasma membrane vesicles revealed that proton pumping was activated by an order of magnitude when a terminal fragment was released by trypsin or chymotrypsin (Palmgren, Larsson, & Sommarin, 1990), and subsequent work revealed that the portion of the pump that had been removed by proteases derived from the C‐terminus, which suggested that this part of the pump could serve as a regulatory autoinhibitory domain (Palmgren, Sommarin, Serrano, & Larsson, 1991).

With the advent of techniques of molecular biology, sequences of plasma membrane H^+^‐ATPase genes were cloned from Arabidopsis thaliana (Harper, Surowy, & Sussman, 1989, Harper, Manney, DeWitt, Yoo, & Sussman, 1990; Pardo & Serrano, 1989), Nicotiana plumbaginifolia (Boutry, Michelet, & Goffeau, 1989), and *Lycopersicum esculentum* (Ewing, Wimmers, Meyer, Chetelat, & Bennett, 1990). This definitely showed the plant plasma membrane H^+^‐ATPase to be evolutionarily related not only to its fungal counterparts but also to P‐type ATPases of animals. Soon thereafter, it became apparent that in each plant investigated, a multigene family of around 10 plasma membrane H^+^‐ATPase genes is present (reviewed in Arango, Gévaudant, Oufattole, & Boutry, 2003; Sondergaard, Schulz, & Palmgren, 2004). Different plasma membrane H^+^‐ATPase genes vary in their spatiotemporal expression patterns and often have specialized physiological roles in the plant body. In A. thaliana, the two most highly expressed isoforms are *Autoinhibited H* ^*+*^ *‐ATPase1* and *2* (*AHA1* and *AHA2*). Under optimal laboratory growth conditions, plants carrying deletions in either gene appear normal, but the homozygous double mutations cause embryo lethality (Haruta et al., 2010). This demonstrates that in plants, like in yeast, the plasma membrane H^+^‐ATPase is essential for cell function.

The heterologous expression of the plant H^+^‐ATPase in cells of S. cerevisiae with reduced expression or devoid of endogenous plasma membrane H^+^‐ATPase proved to be a very convenient tool for producing recombinant plant plasma membrane H^+^‐ATPases in large quantities (de Kerchove d'Exaerde et al., 1995; Villalba, Palmgren, Berberián, Ferguson, & Serrano, 1992). With these systems in hand, it was possible to carry out detailed structure–function analysis by mutagenesis of plant plasma membrane H^+^‐ATPases. It has been used later on by the group of Goffeau to study thermophilic H^+^‐ATPases from Archea (Morsomme et al., 2002).

Using the technology of heterologous expression of plant plasma membrane H^+^‐ATPase in yeast, it could be confirmed by mutagenesis that the C‐terminal region of the protein serves as an autoinhibitory domain (Palmgren & Christensen, 1993). Thus, whereas the full‐length A. thaliana *AHA2* gene could not complement functionally the loss of endogenous yeast plasma membrane H^+^‐ATPase *PMA1*, a mutated *AHA2* gene encoding a protein truncated by 92 C‐terminal residues complemented a *pma1* null mutant. Subsequent mapping by mutagenesis of the C‐terminal domain revealed that it contained two autoinhibitory regions and thus was considerably larger and more complicated in structure than the short C‐terminal autoinhibitory stretch in the yeast H^+^‐ATPase (Axelsen, Venema, Jahn, Baunsgaard, & Palmgren, 1999; Regenberg, Villalba, Lanfermeijer, & Palmgren, 1995).

Mutant screening further identified residues in plant plasma membrane H^+^‐ATPases that were outside of the C‐terminal domain but when they became mutated also gave rise to an activated enzyme (Morsomme et al., 1996; Morsomme, Dambly, Maudoux, & Boutry, 1998). These residues were scattered throughout the primary structure of the pump but were predicted in the 3D structure to form a possible intramolecular receptor for the C‐terminal regulatory domain.

Another feature of the C‐terminal domain of the plant pump that became apparent was that it interacted with activating 14‐3‐3 regulatory proteins (Jahn et al., 1997), that the interaction between the H^+^‐ATPase and 14‐3‐3 protein produced a binding site for the fungal toxin fusicoccin (Baunsgaard et al., 1998; Piotrowski, Morsomme, Boutry, & Oecking, 1998), and that binding of 14‐3‐3 protein to the H^+^‐ATPase required phosphorylation of the penultimate residue of the H^+^‐ATPase (always a threonine) (Fuglsang et al., 1999; Maudoux et al., 2000; Svennelid et al., 1999). Later, it was realized that also the N‐terminal domain is taking part the in regulation of the plant plasma membrane H^+^‐ATPase (Ekberg, Palmgren, Veierskov, & Buch‐Pedersen, 2010). For recent updates on the regulation of the plant plasma membrane H^+^‐ATPase by phosphorylation and other factors, the reader is referred to Duby and Boutry (2009), Haruta, Gray, and Sussman (2015), and Falhof et al. (2016).

Site‐directed mutagenesis of the plant plasma membrane H^+^‐ATPase also resulted in the identification of residues in the transmembrane domain that influence proton pumping. A single proton acceptor‐donor was found to be Asp‐684 (AHA2 numbering) in transmembrane segment 6, whereas the positively charged Arg‐655 in the neighboring transmembrane segment 5 appeared to regulate pumping albeit it was not essential (Buch‐Pedersen et al., 2003a). Based on this work, a model was proposed in which a role for the conserved Arg‐655 is to move in during pumping and provide positive charge to the proton binding site, possibly by polarizing the Asp‐684 side chain and modulating its p*K*_a_, in this way promoting release of the bound H^+^ (Buch‐Pedersen & Palmgren, 2003b). The yeast expression system allowed for production of amounts of AHA2 enzyme sufficient for crystallographic studies. The final crystal structure calculated at 3.6 Å resolution was a major step forward (Pedersen, Buch‐Pedersen, Morth, Palmgren, & Nissen, 2007) and confirmed earlier predictions on the mechanism of H^+^ transport by the pump and proved the plant plasma membrane H^+^‐ATPase to be very similar in structure to other P‐type ATPases such as the Ca^2+^‐ATPase and Na^+^/K^+^‐ATPase (reviewed in Buch‐Pedersen, Pedersen, Veierskov, Nissen, and Palmgren (2009). Unfortunately, neither the N‐ nor the C‐terminal domains were ordered in the structures, so we still do not know how they interfere with pump activity, for which purpose a structure of the pump in its autoinhibited state is required. What is also lacking is high‐resolution 3D structures of both plant and fungal plasma membrane H^+^‐ATPases, which would allow for determining in more detail the H^+^ transport mechanism. These are important goals for the future.

As is evident from this work, many scientists have contributed to the history of the discovery of the plasma membrane H^+^‐ATPase, and often the same discoveries were made by different laboratories at about the same time. We apologize for not having been able to cite all those who have been part of the journey and will end this historical treatise by a relevant quote of Efraim Racker:

Perhaps scientists are too much concerned with giving and receiving credits. The research work in biochemistry in the twentieth century is probably like the building of cathedrals in the Middle Ages. It is the work of many, and the identity of those who participated in their creation is a matter of little consequence and will soon be forgotten. Racker (1976)
